# Spondylodiscite mycosique

**DOI:** 10.11604/pamj.2015.22.31.7831

**Published:** 2015-09-15

**Authors:** Rachid Ammor, Assou Ajja

**Affiliations:** 1 Neurosurgery, Military Hospital My Ismail, Meknes, Morocco

**Keywords:** Douleur lombaire, spondylodiscite, mycose, Lower back pain, spondylitis, mycosis

## Image en medicine

Il s'agit d'un patient de 47 ans, ayant comme antécédent une ponction biopsie prostatique il ya 3 mois. Le patient consulte pour des douleurs du bas du dos d'aggravation rapidement progressive avec apparition depuis une semaine des lombosciatalgies bilatérales mal systématisées. L'examen clinique trouve un patient apyrétique, sans déficit sensitivomoteur ni troubles sphinctériens. L'examen du rachis trouve uns syndrome rachidien lombaire en regard de L3-L4et L5. La radiographie du rachis lombaire a montré un pincement discal en L3-L4 avec érosion des plateaux vertébraux adjacents (A). L'IRM a objectivé la spondylodiscite L3-L4 (B). Une ponction biopsie discale a posé le diagnostic de spondylodiscite mycosique à candida albicans. Les investigations paracliniques n'ont pas objectivé d'immunodépression. Le patient a été mis sous traitement antimycosique avec contention par corset; l’évolution clinique et biologique était favorable. Les spondylodiscites mycosiques sont rares (1 à 2% des spondylodiscites infectieuses) et sont l'apanage des sujets immunodéprimés, sous traitement antibiotique prolongé ou ayant un cathéter d'alimentation parentéral.

**Figure 1:**
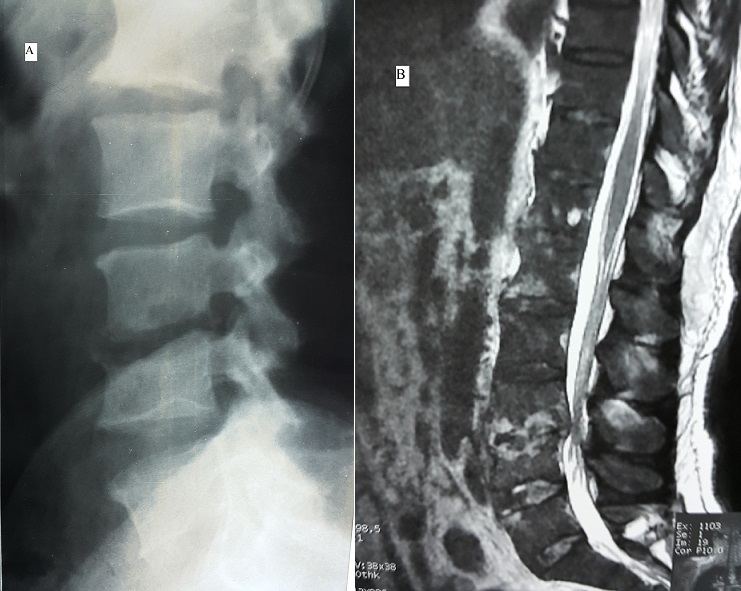
(A) radiographie rachis lombaire de profil montrant le pincement discal en L3-L4 avec érosion des plateaux vertébraux; (B)IRM lombaire, coupe sagittale en séquence pondérée T2 montrant l'hyper signal des plateaux vertébraux en L3-L4avec perte de la limite vertèbre disque

